# Transplacental Transport of Artificial Sweeteners

**DOI:** 10.3390/nu15092063

**Published:** 2023-04-25

**Authors:** Magnus Leth-Møller, Christina Søndergaard Duvald, Sofie Stampe, Eva Greibe, Elke Hoffmann-Lücke, Michael Pedersen, Per Glud Ovesen

**Affiliations:** 1 Department of Obstetrics and Gynecology, Aarhus University Hospital, Palle Juul-Jensens Boulevard 99, 8200 Aarhus Nord, Denmark; sofiestampe@clin.au.dk; 2 Comparative Medicine Lab, Department of Clinical Medicine, Aarhus University, Palle Juul-Jensens Boulevard 11, 8200 Aarhus Nord, Denmark; christina.duvald@clin.au.dk (C.S.D.); michael@clin.au.dk (M.P.); 3 Department of Clinical Medicine, Health, Aarhus University, Palle Juul-Jensens Boulevard 99, 8200 Aarhus Nord, Denmark; evagreib@rm.dk (E.G.); elkehoff@rm.dk (E.H.-L.); 4 Department of Clinical Biochemistry, Aarhus University Hospital, Palle Juul-Jensens Boulevard 99, 8200 Aarhus Nord, Denmark; 5 Steno Diabetes Center Aarhus, Aarhus University Hospital, Palle Juul-Jensens Boulevard 11, 8200 Aarhus Nord, Denmark

**Keywords:** artificial sweeteners, pregnancy, overweight, childhood overweight, fetal programming

## Abstract

The prevalence of obesity is increasing, and the origins of obesity and metabolic dysfunction may be traced back to fetal life. Currently, overweight pregnant women are advised to substitute sugar-sweetened beverages with diet drinks containing artificial sweeteners. Recent evidence suggests that the consumption of artificial sweeteners during pregnancy increases the risk of obesity in the child, but the mechanism is unknown. We hypothesized the transportation of artificial sweeteners across the placenta into the fetal circulation and the amniotic fluid. We included 19 pregnant women who were given an oral dose of acesulfame, cyclamate, saccharin, and sucralose immediately before a planned caesarean section. Nine women were included as controls, and they refrained from an intake of artificial sweeteners. The maternal and fetal blood and amniotic fluid were collected during the caesarean section, and concentrations of artificial sweeteners were measured using mass spectrometry. We found a linear relationship between the fetal plasma concentrations of artificial sweeteners and the maternal plasma concentrations, with adjusted coefficients of 0.49 (95% CI: 0.28–0.70) for acesulfame, 0.72 (95% CI: 0.48–0.95) for cyclamate, 0.51 (95% CI: 0.38–0.67) for saccharin, and 0.44 (95% CI: 0.33–0.55) for sucralose. We found no linear relationship between amniotic fluid and fetal plasma concentrations, but there were positive ratios for all four sweeteners. In conclusion, the four sweeteners investigated all crossed the placenta and were present in the fetal circulation and amniotic fluid.

## 1. Introduction

The prevalence of overweight and obesity among women of childbearing age is increasing. A 2017 report from the Danish National Board of Health showed that, among Danish females aged 25–34 years, 37% are overweight (BMI ≥ 25 kg/m^2^), and 15% are obese (BMI ≥ 30 kg/m^2^) [1]. This tendency is paralleled by an increased prevalence of gestational diabetes mellitus in Denmark across all age groups [2].

In response to these disturbing figures, several health authorities have released advice to substitute sugary drinks with artificially sweetened drinks [3]. However, recent evidence suggests that consumption of artificial sweeteners (ASs) during pregnancy increases the risk of obesity in the child [4,5].

Artificial sweeteners are synthetic food additives that are widely used to lower caloric intake, and diet drinks are major contributors to their consumption. They replace sugar in diet products with sweetening intensities 200–600 times that of tabletop sugar [6]. They are generally considered safe; however, there is ongoing discussion about their efficacy in weight loss [7] and their role in increasing the risk of type 2 diabetes [8]. Artificial sweeteners have recently been suspected as substances capable of altering metabolic programming in utero [9,10]. Thus, Azad et al. [4] and Zhu et al. [5] found an increased risk of excess weight in the children of women with a high intake of artificial sweeteners during pregnancy.

These findings indicate evidence of transplacental fetal exposure to ASs. To address this concern, we investigated the transplacental transport of ASs into the fetal circulation with subsequent fetal accumulation. In the fetus, the amniotic fluid is mostly derived from fetal urination, and the amniotic fluid is primarily eliminated through fetal swallowing [11]. The fetal metabolism of ASs is unknown; however, it could be speculated that AS are excreted unmetabolized (as they mostly are in the adult [6]) in the urine/the amniotic fluid, swallowed by the fetus, and then reabsorbed to the fetal circulation system. Hence, an increasing concentration of AS may accumulate in the fetus.

The aim of this study was to investigate the transplacental transport of five artificial sweeteners.

## 2. Materials and Methods

We conducted a controlled, open-labelled, clinical investigation with imbalanced allocation of intervention (3:1). We aimed for 30 in the intervention group and 10 controls. We included the controls first. Initially, we included patients for intervention in three groups. Group 1: Women with diabetes (both pre-gestational and gestational). Group 2: Women with IUGR children (defined as having estimated fetal weight < −15% of expected). Group 3: Healthy women. After inclusion had begun, all three intervention groups were pooled due to low numbers in groups 1 and 2 (see Section 3.1). Due to lack of previous research on the subject, it was not possible to perform a power calculation.

Eligible participants were pregnant women, aged 18 or older, who understood Danish, and who underwent a planned caesarean section (C-section). Exclusion criteria were vaginal delivery or acute C-section, and significant co-morbidities such as active inflammatory bowel disease or previous gut-altering surgery (e.g., resection of intestines or gastric bypass). We asked women to participate between June 2019 and March 2020 at Aarhus University Hospital, Denmark, when they came for a pre-operation informational consultation two to three days before their planned C-section.

Participants allocated to the intervention were given 250 mL of unsweetened, blackcurrant-flavored juice, sweetened with 85 mg acesulfame K, 100 mg aspartame, 60 mg cyclamate, 20 mg saccharin, and 75 mg sucralose. These sweeteners were chosen because they are widely used in diet products, and are approved by the European Food Safety Authority. The amounts were chosen to achieve measurable plasma concentrations, and with consideration of the Acceptable Daily Intake (ADI). Participants were instructed to drink it two hours before the C-section. Most sweeteners are rapidly absorbed and metabolized, but women were not allowed to consume drinks less than two hours before surgery. Both participants in the intervention group, and in the control groups, were asked to refrain from intake of diet drinks for 48 h before the C-section. Since most sweeteners are eliminated within 24 h [6], the period of forty-eight hours was deemed an appropriate amount of time to eliminate residual artificial sweeteners ingested prior to the study. 

**Figure 1:**
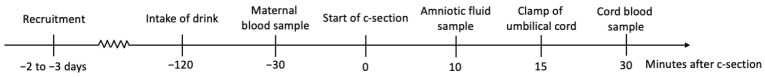
Conceptualization of the designed flow of intake and sampling of artificially sweetened drink.

The investigator obtained maternal blood samples in an EDTA tube immediately before the C-section. During the C-section, the surgeon obtained amniotic fluid using a syringe, when possible by inserting the needle into the amnion and aspirating fluid. After delayed cord clamping, the umbilical cord was cut and we obtained cord-blood samples in EDTA tubes. Sampling flow is illustrated in Figure 1.

The blood and amniotic fluid were centrifuged at 2000 g at 4 °C for 10 min, and the supernatant was transferred to freeze-tubes and stored at −80 °C until analysis. Amniotic fluid was inspected for blood and meconium contamination by visual inspection after centrifugation.

The method for measuring the sweeteners is described elsewhere [12]. In brief, concentrations of the ASs were measured by High-Performance Liquid Chromatography with Tandem Mass Spectrometry (LC-MS/MS). The liquid chromatography was carried out on the Agilent 1290 Infinity Series system (Agilent Technologies, Glostrup, Denmark), and mass spectrometric detection was carried out on the Agilent 6470 Triple Quad mass spectrometer (Agilent Technologies, Glostrup, Denmark). This was equipped with an electrospray ionization source. Analytical separation was performed on a Luna Omega C18 column (dimensions: 2.1 mm × 50 mm, 1.6 µm C18) (Phenomenex, Copenhagen, Denmark) at a temperature of 30 °C, which was controlled by a column heater. Aspartame was not reported since it is metabolized in the gut lumen to its metabolites [6], and therefore does not enter the circulation.

The correlation between maternal and fetal plasma concentrations of ASs were considered the primary outcome, and the correlation between amniotic fluid concentrations and fetal plasma concentrations the secondary outcomes. All analyses were performed after trial completion.

The study was approved by the Central Denmark Region Ethical Committee with no. 1-10-72-76-19, and registered on clinicaltrials.gov with id NCT03954418.

### Statistics

We described participant demographics, time intervals from drink-to-sample, and sample-to-sample intervals, using medians with 1st and 3rd quartiles.

Fetal and maternal plasma and amniotic fluid concentrations were calculated using summary statistics and reported as means with 95% confidence intervals (CI). We calculated feto–maternal plasma concentration ratios and amniotic fluid–fetal plasma concentration ratios.

Crude correlations between fetal and maternal plasma concentrations, and between amniotic fluid and fetal plasma concentrations, were computed using linear regression. Adjusted correlation coefficients were calculated using multiple linear regression. We adjusted for maternal weight, birthweight, and time from maternal sample to umbilical clamp for maternal–fetal plasma correlation, and time from amniotic sample to umbilical sample in the fetal–amniotic correlation.

Normality was evaluated using histograms and Q-Q plots. Statistical significance was evaluated using 95% confidence intervals. Statistical analyses were performed using Stata Statistical Software: Release 16. 2019. College Station, TX, USA: StataCorp LLC.

## 3. Results

### 3.1. Participants

Forty-five women were invited to participate, of whom thirty-five accepted. In the control group, one participant was excluded because of an intake of an AS on the morning of delivery. Amniotic fluid sampling failed for one control, but the participant was not excluded. In the intervention group, four were excluded because they did not ingest the provided drink, or because of emesis between intake and C-section. One participant gave birth vaginally before the planned C-section. One participant was excluded because of reverse sampling order (fetal blood sampled before maternal). We planned to include women in three groups: healthy women, women with diabetes, and women with IUGR children, but due to a low number of women with diabetes (two with gestational diabetes; one with type 1 diabetes, and one with type 2 diabetes), and women with growth-restricted fetuses (n = 1), we pooled the three groups. In total, there were nine controls and nineteen in the intervention group.

The study was stopped prior to reaching the planned sample size to ensure completion within the available timeframe of the project.

Reasons for declining participation were anxiety, avoidance of ASs, and participation in other trials.

Participants in the intervention group had a median weight of 80.0 kg (from 1st to 3rd quartile (IQR) 71.3–85.3) at delivery, and had a median pre-pregnancy BMI of 23.4 kg/m^2^ (IQR: 20.3–25.6). Baseline demographics for participants are shown in Table 1. We observed no significant differences between the intervention group and controls.

Median time interval from intake of an AS to maternal blood sample was 223 min (IQR: 142–273), and from maternal blood sample to umbilical clamp was 30 min (IQR: 26–36) (Table 2).

### 3.2. Association between Maternal and Fetal Plasma Concentrations

**Figure 2:**
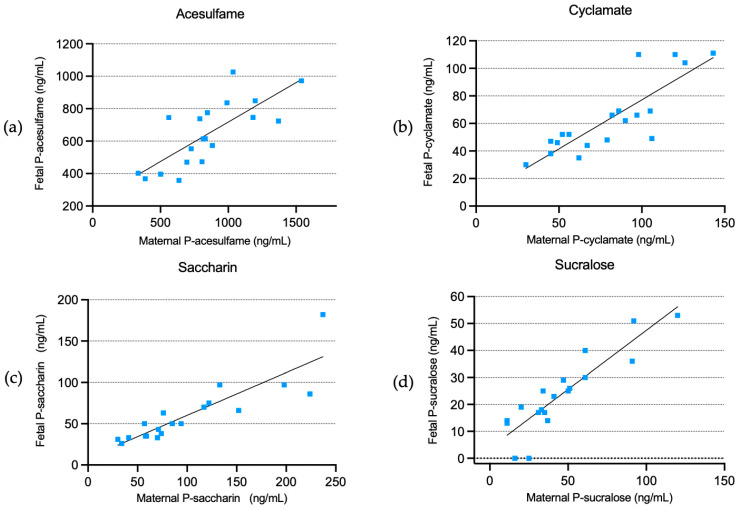
Fetal plasma concentrations of acesulfame (**a**), cyclamate (**b**), saccharin (**c**), and sucralose (**d**) in relation to maternal plasma concentrations.

We found that fetal plasma concentrations had a significant, positive, linear dependency on the maternal plasma concentrations of all four artificial sweeteners, with adjusted coefficients of 0.49 (CI: 0.28–0.70) for acesulfame, 0.72 (CI: 0.48–0.95) for cyclamate, 0.51 (CI: 0.38–0.67) for saccharin, and 0.44 (0.33–0.55) for sucralose (Figure 2 and Table 3).

Additionally, there were positive ratios between fetal and maternal plasma concentrations of 0.80 (CI: 0.70–0.90) for acesulfame, 0.80 (CI: 0.72–0.89) for cyclamate, 0.65 (CI: 0.56–0.73) for saccharin, and 0.57 (CI: 0.42–0.73) for sucralose.

In the control group, two out of nine participants had maternal plasma concentrations of acesulfame above the lower limit of quantification (LLOQ), with a mean of 0.01 ng/mL (CI: −0.07–0.09). Six controls had measurable concentrations of saccharin, with a mean of 3.78 ng/mL (CI: 0.89–6.67). For cyclamate and sucralose, all controls had plasma concentrations below LLOQ.

The fetal plasma concentrations of acesulfame were >LLOQ in three controls, with a mean of 2.90 ng/mL (CI: −4.64–10.45). Two participants had measurable plasma concentrations of cyclamate, with a mean of 3.87 ng/mL (CI: 1.17–6.57), and four had plasma concentrations of saccharin >LLOQ, with a mean of 2.67 ng/mL (CI: −0.13–5.47). No controls had plasma concentrations of sucralose >LLOQ (Table 4).

### 3.3. Association between Amniotic Fluid Concentrations and Fetal Plasma Concentrations

Acesulfame, cyclamate, and saccharin were present in the amniotic fluid in all participants in the intervention group, with mean concentrations of 494.75 ng/mL (CI: 230.53–758.97), 17.30 ng/mL (CI: 10.12–24.48), and 61.32 ng/mL (CI: 42.03–80.61), respectively. Sucralose was above the LLOQ in four cases, with mean amniotic concentrations of 12.20 ng/mL (CI: 0.08–24.33) and a range of 0–70.53 ng/mL (see Table 3, range not shown).

**Figure 3:**
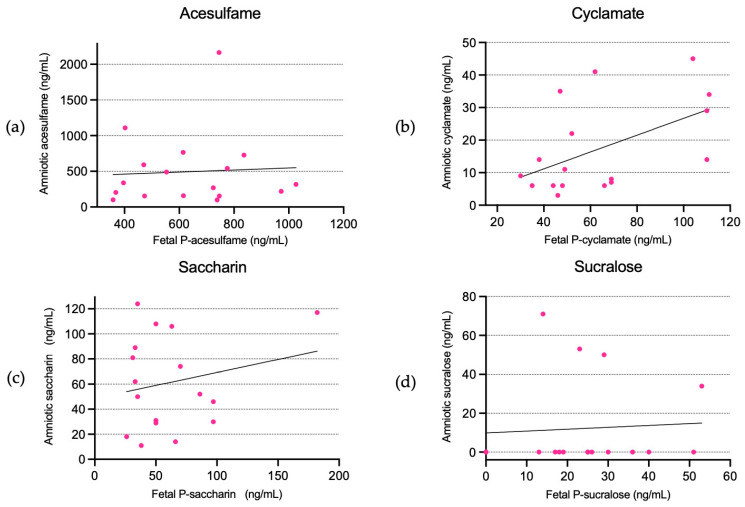
Amniotic fluid concentrations of acesulfame (**a**), cyclamate (**b**), saccharin (**c**), and sucralose (**d**) in relation to fetal plasma concentrations.

There were no linear associations between amniotic fluid concentrations and fetal plasma concentrations, except a weak association for cyclamate in the unadjusted model (b: 0.25 (CI: 0.01–0.49)) (Figure 3).

There were amniotic–fetal ratios for acesulfame, cyclamate, saccharin, and sucralose of 0.83 (CI: 0.33–1.32), 0.28 (CI: 0.16–0.39), 1.09 (CI: 0.62–1.57), and 0.66 (CI: −0.14–1.45), respectively.

In the control group, three out of eight participants had acesulfame concentrations in the amniotic fluid >LLOQ, with a mean of 26.65 ng/mL (CI: −20.5–73.8); note that we failed to obtain an amniotic fluid sample in one control. Seven controls had amniotic fluid saccharin >LLOQ, with a mean of 2.94 ng/mL (CI: 0.99–4.89). One control had cyclamate >LLOQ, with a concentration of 11.98 ng/mL. No controls had sucralose >LLOQ (Table 4).

## 4. Discussion

### 4.1. Key Findings

This study shows that the ASs, acesulfame, cyclamate, saccharin, and sucralose readily cross the placental barrier and enter the fetal circulation, and that these artificial sweeteners are present in the amniotic fluid.

### 4.2. Feto-Maternal Correlation

We found a positive, linear correlation between maternal and fetal plasma concentrations, and for all sweeteners with adjusted coefficients varying from 0.45 to 0.69, and with ratios ranging from 0.57 to 0.80. This is suggestive of a passive diffusion of the sweeteners across the placenta. There are, to our knowledge, no previous studies to support this theory, but the sweeteners are mostly excreted unmetabolized in the urine [6].

### 4.3. Amniotic Fluid

Since most sweeteners are excreted by the kidneys unmetabolized, it is likely that once the sweeteners have entered the fetal circulation they are excreted through the fetal kidneys into the amniotic fluid.

With increasing time from intake of acesulfame and saccharin to maternal sampling we observed decreasing maternal plasma concentrations (Table S1 and Figure S1 (Supplementary Materials)), probably due to renal excretion in this time period. Contrary to these findings, amniotic fluid concentrations significantly increased with increasing time from maternal intake to amniotic fluid sampling for acesulfame and cyclamate. For saccharin and sucralose, we also found a non-significant trend for increasing concentrations with increasing time (Table S2 and Figure S2).

We suggest that ASs ingested by women pass the placenta to the fetal circulation, where they are excreted renally to the amniotic fluid and then reabsorbed by the fetus, thereby accumulating in the feto–amniotic circulation. This has not been investigated in ASs, but it has been investigated in the antiretroviral medication zidovudine and lamivudine. They, in the same way as ASs, cross the placenta freely and are excreted renally. Chappuy et al. found an increased ratio of drug concentration between the amniotic fluid and cord and the maternal blood, suggesting an accumulation in the feto–amniotic circulation [13].

Another possible explanation is a delay in the chain of transfers–from maternal intestine, to maternal circulation, to fetal circulation, to amniotic fluid–and that we have simply not observed a long enough time period to witness the decrease in amniotic fluid concentration. Further research is needed to answer these questions.

Only four of nineteen participants in the intervention group had measurable sucralose in the amniotic fluid, whereas they all had measurable concentrations of the other sweeteners. The LLOQ for the sucralose analysis was 10 ng/mL, rather than 1 ng/mL, as is the case for the other sweeteners. This, combined with the kinetics of sucralose (which is mainly excreted in the faeces, and only to a lesser extent the urine), could cause the amniotic fluid concentrations to be below 10 ng/mL [14].

### 4.4. Potential Mechanisms and Explanations

Other studies have investigated the effects of artificial sweeteners consumed during pregnancy on child BMI; for example, Azad et al. found an adjusted odds ratio (OR) of 2.19 (95% CI: 1.23–3.88) for overweight in the 1-year-old offspring of women with an intake of ≥1 serving per day of artificially sweetened soft-drink, compared to no intake [4]. Interestingly, Zhu et al. did not find this association among 1-year-olds in a population of women with gestational diabetes, but did find a relative risk (RR) of 1.93 (95% CI: 1.24–3.01) for overweight in the 7-year-old offspring of women with a daily intake of ≥1 diet drink, compared to no consumption [5].

Other than metabolic changes, pre-term delivery [15,16], forearm fractures [17], and asthma and allergic rhinitis [18] in the offspring have been linked to high intake of ASs during pregnancy.

The studies by Azad et al. and Zhu et al. suggest that ASs have an impact on the developing fetus, but provide no mechanistical explanation; however, proposals have pointed to a relationship between ASs and altered metabolic programming during fetal life. ASs can activate sweet-taste receptors in the small intestine of rats [9,19], and acesulfame has been found to affect sweet-taste sensation and preferences in the offspring [20].

It is possible that part of the effect on offspring metabolism is mediated by breastfeeding. Most likely, women with a high intake of diet drinks may continue this intake during breastfeeding. We have, in a previous study, shown that ASs ingested by breastfeeding women are transferred to the milk, possibly affecting the nursing infant [21].

### 4.5. Strengths and Limitations

A major strength of this study is the controlled, standardized design, whereby both the amount ingested, and the timing, are recorded, and plasma concentrations are objectively measured. Previous studies on the subject have primarily relied on self-reported intake data detailing the number of diet drinks ingested, presenting possible recall biases [4,5]. Furthermore, many may ingest ASs without knowing, e.g., we observed ASs in six out of nine controls who had refrained from the intake of diet products for at least 24 h.

Our study might be limited by the open-label, non-randomized design, but we believe that the impact, if present, is minimal. This is due to the objective measurement of plasma and amniotic fluid concentrations of ASs.

Furthermore, we planned to compare women with diabetes and women with IUGR children, however, due to very low numbers of these groups being eligible (four with diabetes, and one with growth restriction), we pooled all participants in the intervention group. We believe that both increased and decreased transport of ASs across the placenta in women with diabetes, or with growth-restricted fetuses due to the dysfunction of the placenta, is possible [22,23]. This could potentially lead to bias due to both under- and over-estimation in this study, but it is still unknown what role placental function plays.

We included healthy women in one group, however, because these women also underwent planned C-sections, one could say that they are not truly healthy, namely because was an indication for the C-section. We tried to limit this bias by excluding women with, for example, gut-altering surgery or inflammatory bowel disease, as these could impact the absorption of ASs. Other indications for C-section are fetal malpresentation, previous C-section, and maternal request. We do not believe that these conditions would affect the absorption or distribution of ASs.

### 4.6. Sources of Error

When sampling amniotic fluid, we inserted a needle through the fetal membranes to draw fluid. When doing so, there is a risk of the amnion bursting and the amniotic fluid being mixed with maternal blood from the surgical site. To address this, the investigator noted whether amniotic fluid was contaminated with blood or meconium by visual inspection of the centrifuged sample. Thirteen in the intervention group had contaminated amniotic fluid, and four did not. We observed no statistically significant difference in the concentrations of any of the four sweeteners between contaminated and non-contaminated amniotic fluid samples (Table S3). No samples had signs of meconium contamination (green or brown coloring identified by visual inspection).

When collecting umbilical cord samples, we strived to collect venous blood, however, in some cases, venous and arterial bloods were mixed. It is possible that the concentrations of ASs vary between arterial and venous umbilical cord blood, and that the ratio between the two probably varies in our samples. Future studies should investigate the difference between arterial and venous blood.

There was significant variation in the intervals from intake of the drink to sampling due to logistical elements. This is controlled for in the adjusted beta coefficients.

To be certain that the measured ASs stemmed from the intervention, we included controls in our study who refrained from intake of ASs. We found ASs in six maternal, four fetal, and seven amniotic fluid samples (Table 4). The sweeteners have relatively short half-lives, and the small concentrations observed are most likely due to the participants unknowingly ingesting ASs in the days before C-section. Saccharin was found in seven amniotic fluid samples, but only four fetal plasma samples; this raises the suspicion of the accumulation in the amniotic fluid (Table 4).

Since ASs were detected in the control group, we cannot rule out that participants in the intervention group also had sweeteners in their circulation prior to intake of the provided drink. By investigating the correlation between maternal and fetal plasma concentrations, we minimized the impact on the results.

We investigated the properties of sweeteners in late pregnancy, but the developing fetus is constantly changing, and both the renal system and amniotic fluid change over the course of pregnancy. Therefore, the properties we observed might not be applicable in early- or mid-pregnancy.

### 4.7. Summary and Clinical Implications

Previous studies have shown an association between the high intake of diet drinks and future risk of excess weight in offspring [4,5]. We are the first to show that ASs do indeed cross the placental barrier, and thereby have the possibility of affecting the developing fetus. Further research is warranted to determine whether the sweeteners alter the fetal programming or metabolism.

Offspring of women with overweight and/or diabetes, tend to have a higher risk of becoming overweight themselves. The current advice for pregnant women with overweight and pregnant women with diabetes is to substitute sugary drinks; this might, therefore, put already at-risk children at further risk of becoming overweight if the sweeteners alter metabolic programming.

## 5. Conclusions

In conclusion, we found that artificial sweeteners ingested by pregnant women are present in both the fetal circulation and the amniotic fluid.

## Figures and Tables

**Table 1:** Baseline demographics of participants. All values are medians (from 1st to 3rd quartile).

Characteristics	Intervention (n = 19)	Controls (n = 9)
Weight at delivery (kg)	80.0 (71.3–85.3)	91.7 (84.4–95.0)
Pre-pregnancy BMI (kg/m^2^)	23.4 (20.3–25.6)	25.3 (20.5–27.0)
Gestational age (days)	271 (266.5–271.5)	271.0 (266.0–272.0)
Placental weight (g)	580.0 (559.0–667.5)	500.0 (495.0–650.0)
Birthweight (g)	3520 (3200–3790)	3410 (3080–3610)
Child length (cm)	51.0 (50.0–52.5)	51.0 (51.0–51.0)
Head circumference (cm)	35.0 (34.0–36.0)	35.0 (34.0–36.0)
Abdominal circumference (cm)	33.0 (32.3–34.0)	32.0 (31.0–34.0)

**Table 2:** Time intervals between intake of artificial sweeteners and sampling. All values are medians (from 1st quartile to 3rd quartile).

Time Intervals of Sampling	Minutes
Intake to maternal sample	223 (142–273)
Intake to umbilical clamp	274 (166–319)
Maternal sample to umbilical clamp	30 (25–36)
Intake to amniotic sample	274 (165–317)
Amniotic sample to umbilical clamp	2 (1–3)

**Table 3:** Correlations between maternal and fetal plasma concentrations (top), and amniotic and fetal concentrations (bottom), in the intervention group.

Feto-Maternal Correlation	Fetal Plasma Conc. (95% CI)	Maternal Plasma Conc. (95% CI)	Crude b (95% CI)	Adjusted ^a^ b (95% CI)	Ratio (95% CI)
Acesulfame (ng/mL)	643.9 (546.3–741.4)	848.5 (695.7–1001.3)	0.49 (0.28–0.70)	0.53 (0.30–0.76)	0.80 (0.70–0.90)
Cyclamate (ng/mL)	63.4 (50.7–76.2)	81.0 (66.0–95.9)	0.72 (0.48–0.95)	0.69 (0.41–0.98)	0.80 (0.72–0.89)
Saccharin (ng/mL)	61.2 (43.5–79.0)	101.8 (71.8–131.7)	0.51 (0.38–0.67)	0.56 (0.38–0.73)	0.65 (0.56–0.73)
Sucralose (ng/mL)	23.6 (16.7–30.5)	45.6 (31.5–59.7)	0.44 (0.33–0.55)	0.45 (0.30–0.59)	0.57 (0.42–0.73)
**Amniotic-fetal** **correlation**		**Amniotic plasma conc.** **(95% CI)**	**Crude b** **(95% CI)**	**Adjusted ^b^** **(95% CI)**	**Ratio** **(95% CI)**
Acesulfame (ng/mL)		494.7 (230.5–759.0)	0.14 (−1.22–1.50)	0.26 (−1.34–1.87)	0.83 (0.33–1.32)
Cyclamate (ng/mL)		17.3 (10.1–24.5)	0.25 (0.01–0.49)	0.21 (−0.09–0.52)	0.28 (0.16–0.39)
Saccharin (ng/mL)		61.3 (42.0–80.6)	0.21 (−0.32–0.73)	0.15 (−0.37–0.67)	1.09 (0.62–1.57)
Sucralose (ng/mL)		12.2 (0.1–24.3)	0.08 (−0.78–0.96)	−0.29 (−1.40–0.83)	0.66 (−0.14–1.45)

Linear regression coefficients of fetal plasma concentrations in relation to maternal plasma concentrations, and amniotic fluid concentrations in relation to fetal plasma concentrations. Concentrations reported as means with 95% CI. ^a^ Adjusted for maternal weight, birthweight, and time from maternal sample to umbilical clamp. ^b^ Adjusted for maternal weight, birthweight, and time from amniotic sample to umbilical clamp.

**Table 4:** Concentrations of artificial sweeteners in the control group.

	Maternal		Fetal		Amniotic Fluid	
	N > LLOQ	Conc.	N > LLOQ	Conc.	N > LLOQ	Conc.
Acesulfame (ng/mL)	2	0.01 (−0.07–0.09)	3	2.90 (−4.64–10.45)	3	26.65 (−20.50–73.80)
Cyclamate (ng/mL)	0	-	2	3.87 (1.17–6.57)	1	*
Saccharin (ng/mL)	6	3.78 (0.89–6.67)	4	2.67 (−0.13–5.47)	7	2.94 (0.99–4.89)
Sucralose (ng/mL)	0	-	0	-	0	-

“N > LLOQ”: Number of controls with values above LLOQ. Mean values are ng/mL with 95% CI. Means are calculated on those with values > LLOQ. * No mean, only one participant.

## Data Availability

Data described in the manuscript, code book, and analytic code will not be made available due to data protection regulations.

## Supplementary Materials

The following supporting information at: https://www.mdpi.com/article/10.3390/nu15092063/s1, Figure S1: Correlation between time and maternal plasma concentrations; Figure S2: Correlation between time and amniotic fluid concentrations Table S1: Maternal plasma concentration over time; Table S2: Amniotic fluid concentration over time; Table S3: Comparison of contaminated and non-contaminated samples.
