# Supplementary material for: Transplacental Transport of Artificial Sweeteners
Source: Nutrients. 2023 Apr 25;15(9):2063. doi: 10.3390/nu15092063 (PMC10181363; doi:10.3390/nu15092063)
Supplement: Supplementary file 1 [file nutrients-15-02063-s001.zip › nutrients-2311189-supplementary.pdf]

nutrients

MDPI

Supplementary Materials

Transplacental Transport of Artificial Sweeteners

Magnus Leth-Møller [1,2,3,*], Christina Søndergaard Duvald [2], Sofie Stampe [1,2], Eva Greibe [3,4], Elke Hoffmann-Lücke [3,4], Michael Pedersen [2] and Per Glud Ovesen [1,3,5,*]

Table S1. Association between maternal plasma concentration (ng/mL) dependent on time from ingestion of drink to maternal blood sample (minutes).

Artificial sweetener	Coefficient (95% CI)
Acesulfame	−2.36 (−3.87 ; −0.84)
Cyclamate	−0.06 (−0.24 ; 0.12)
Saccharin	−0.52 (−0.79 ; −0.26)
Sucralose	−0.10 (−0.27 ; 0.06)

Coefficients are calculated using linear regression and are unadjusted.

Table S2. Association between amniotic fluid concentrations of each sweetener (ng/mL) dependent on time from intake of dink to amniotic fluid sampling (minutes).

Artificial sweetener	Coefficient (95% CI)
Acesulfame	3.87 (1.50 ; 6.23)
Cyclamate	0.10 (0.04 ; 0.17)
Saccharin	0.14 (−0.08 ; 0.36)
Sucralose	0.12 (−0.01 ; 0.25)

Coefficients are calculated using linear regression and are unadjusted.

Table S3. Mean concentration (95% CI) of each sweetener (ng/mL) in amniotic fluid samples in samples recorded as contaminated or non-contamniated.

	Contaminated (n = 8)	Non-contaminated (n = 19)
Acesulfame	360.26 (110.68 ; 609.85)	244.63 (−13.45 ; 502.11)
Cyclamate	12.16 (6.23 ; 18.10)	11.83 (−2.08 ; 25.74)
Saccharin	41.38 (22.36 ; 60.40)	38.27 (−0.03 ; 76.57)
Sucralose	8.28 (−1.63 ; 18.20)	6.26 (−8.54 ; 21.06)

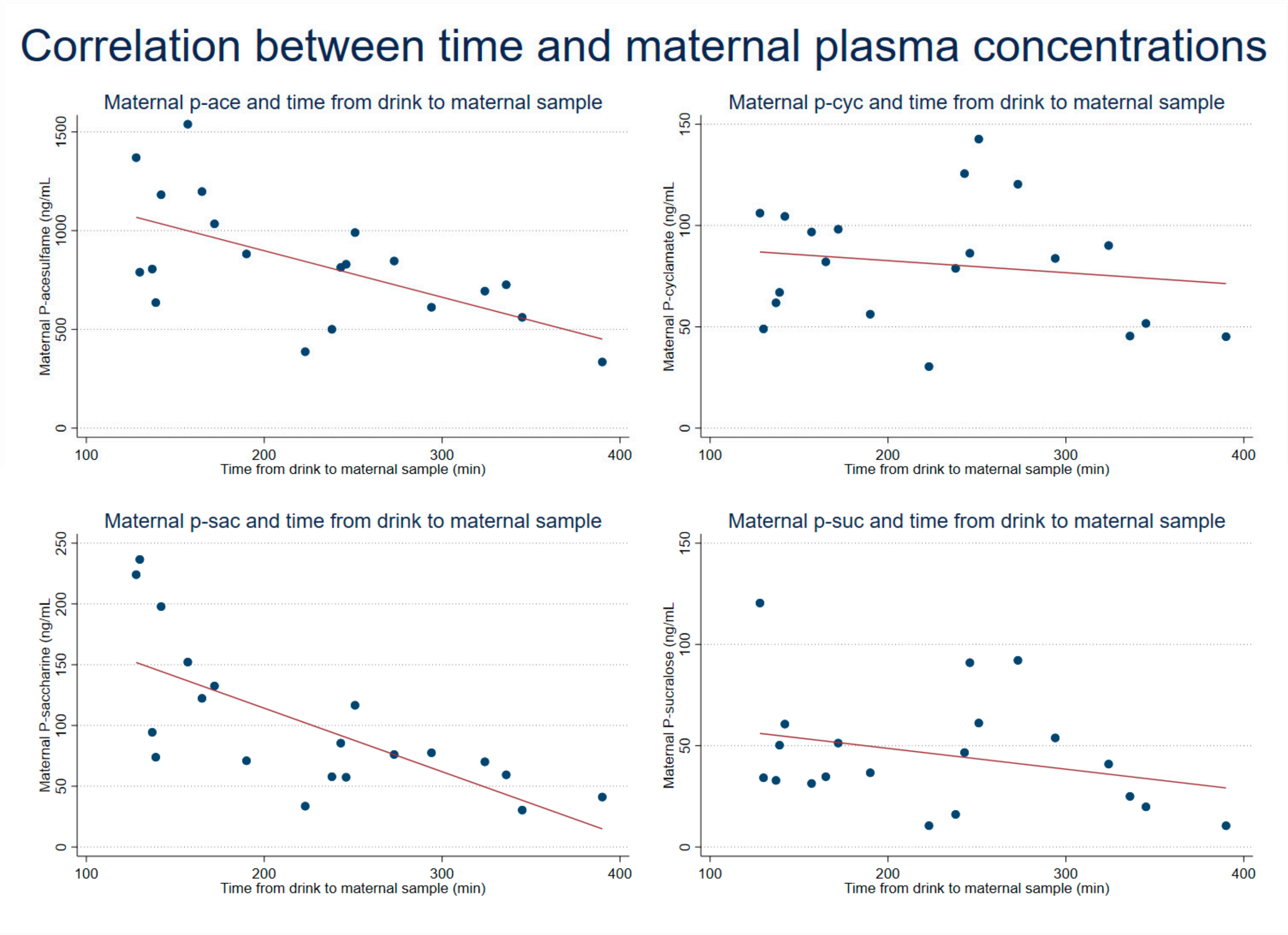

Figure S1. Correlation between time from ingestion of drink and maternal plasma concentration of each sweetener.

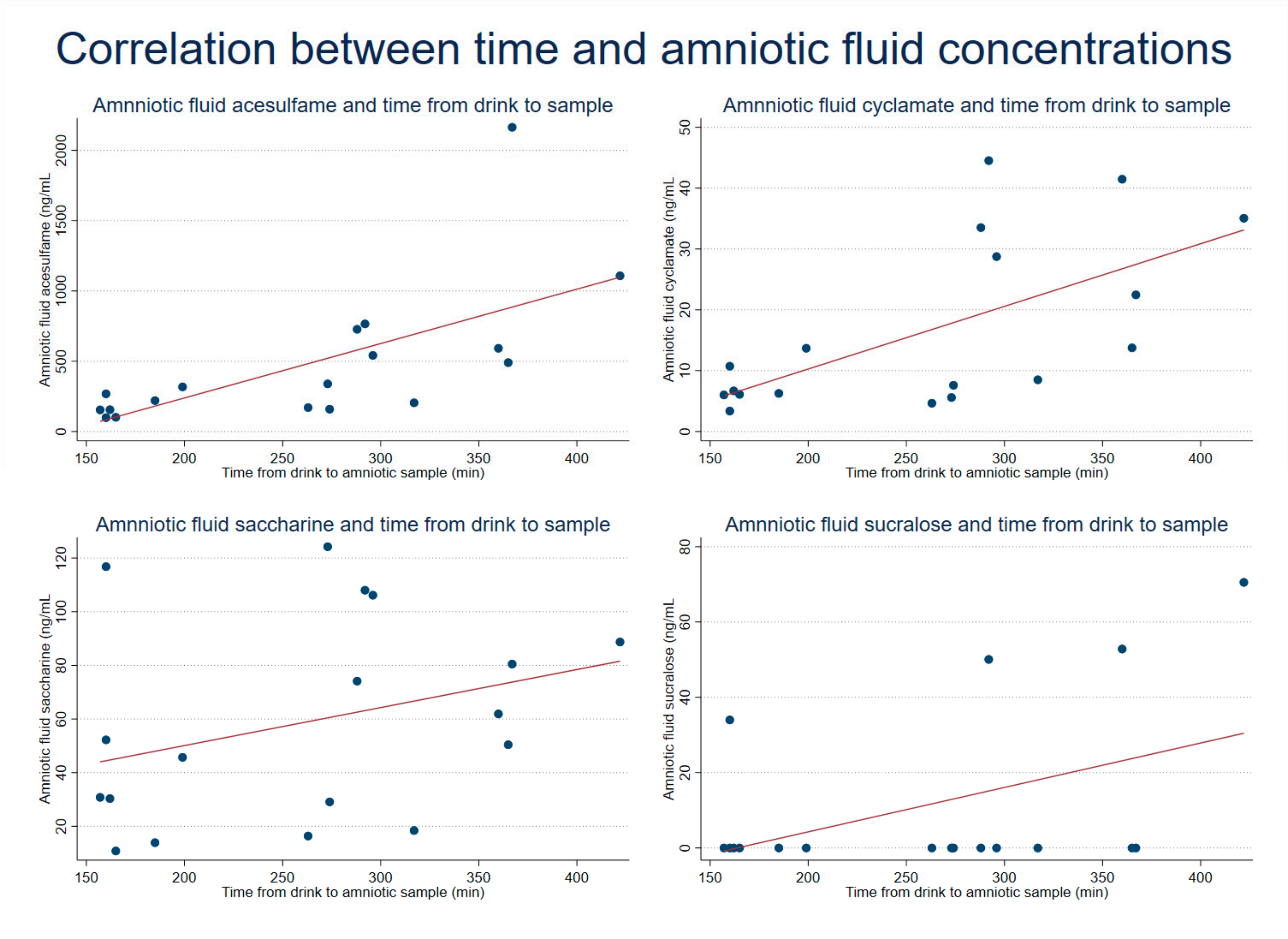

Figure S2. Association between time from ingestion of drink and amniotic fluid sampling and amniotic fluid concentration of each sweetener.
